# Case report: 5-Fluorouracil treatment in patient with an important partial DPD deficiency

**DOI:** 10.3389/fonc.2023.1187052

**Published:** 2023-06-20

**Authors:** Antonin Schmitt, Bernard Royer, Romain Boidot, Joseph Berthier, François Ghiringhelli

**Affiliations:** ^1^ Pharmacy Department, Centre Georges-François Leclerc, Dijon, France; ^2^ Institut National de la Santé et de la Recherche Médicale (INSERM) U1231, University of Burgundy Franche-Comté, Dijon, France; ^3^ Pharmacology and Toxicology Laboratory, Besançon University Hospital, Dijon, France; ^4^ Unit of Molecular Biology, Centre Georges-François Leclerc, Institut de Chimie Moléculaire de l'Université de Bourgogne (ICMUB) Unité Mixte de Recherche (UMR) Centre National de la Recherche Scientifique (CNRS) 6302, Dijon, France; ^5^ Pharmacology and Toxicology Laboratory, Dijon University Hospital, Dijon, France; ^6^ Medical Oncology Department, Centre Georges-François Leclerc, Dijon, France

**Keywords:** uracilemia, DPD deficiency, 5-Fu (5-Fluorouracil), esophageal cancer, therapeutic drug monitoring

## Abstract

Esophageal cancer is a cancer with poor prognosis and the standard 1^st^ line treatment for metastatic or recurrent EC is systemic chemotherapy with doublet chemotherapy based on platinum and 5-fluorouracil (5-FU). However, 5-FU could be a source of severe treatment-related toxicities due to deficiency of dihydropyrimidine dehydrogenase (DPD). In this case report, a 74-year-old man with metastatic esophageal cancer was found to have partial DPD deficiency based on uracilemia measurements (about 90 ng/mL). Despite this, 5-FU was safely administered thanks to therapeutic drug monitoring (TDM). The case report highlights the importance of TDM in administering 5-FU to patients with partial DPD deficiency, as it allows individualized dosing and prevents severe toxicity.

## Introduction

Esophageal cancer (EC) is the seventh most common cancer, accounting for 3.1% of all cancers, and the sixth leading cause of cancer deaths worldwide, accounting for 5.5% of all cases (1). EC is a cancer with a poor prognosis, detected symptomatically in advance stages. Systemic chemotherapy, a doublet chemotherapy based on platinum and 5-fluorouracil (5-FU), is the standard 1^st^ line treatment for patients with metastatic or recurrent EC who have no curative options, despite limited efficacy (2). Moreover, 5-FU could be the source of severe treatment-related toxicities requiring hospitalization and leading to death in 0.5% to 2% of cases (3–5).

The most well-known biochemical cause of intolerance to fluoropyrimidines is deficiency of dihydropyrimidine dehydrogenase (DPD) (6, 7). DPD is defined as the first and rate-limiting enzyme in the catabolic pathway of 5-FU, responsible for more than 80% of 5-FU elimination (8, 9). Partial or complete deficiency in the DPD enzyme has been observed in 3-5% and 0.1% of the general population, respectively (10–12). DPD-deficient patients experience excessive and severe toxicity in the form of neutropenia, diarrhea, mucositis and hand and foot syndrome. DPD deficiency may be investigated by genotyping *DPYD* gene or by phenotyping by means of uracilemia (U) or dihydrouracilemia/U ratio (UH_2_/U).

In France, to date, uracilemia is mandatory before prescription of fluoropyrimidine (uracilemia is not yet mandatory in most countries outside European Union) (13). National institute of cancer in France (INCa) propose that if U < 16 ng/mL patient should be considered with functional DPD, if 16 ≤ U < 150 ng/mL a partial deficiency is suspected and in case of U ≥ 150 ng/mL a total deficiency is suspected. The French recommendations propose to decrease the initial 5-FU dose in case of partial deficiency and to contra-indicate it in case of total deficiency. However, no clear information is given on the extent of dose adaptation in the 1^st^ situation. With regards to UH_2_/U ratio, no consensus exists with regards to the cut-off value for which a patient would be characterized as deficient because of an important heterogeneity in ratio measurements and a poor prognostic value (14–16).

In our center, we routinely monitor 5-FU concentration to adapt doses based on clinical evidence, but also on individual exposure. Recent French recommendations state that exposure after 5-FU infusion of 46h, by mean of Area Under the Curve of the concentrations time course of 5-FU (AUC), should be within 20-30 mg.h/L (17). We report here a case of a metastatic esophageal cancer patient with a partial DPD deficiency (U ≈ 90 ng/mL) for which 5-FU could be administered thanks to Therapeutic Drug Monitoring (TDM).

## Case report

A 74-year-old man was diagnosed in April 2022 with an esophageal adenocarcinoma with bone, liver, lung, and lymph node metastases. As 5-FU is the chemotherapy’s backbone of esophageal cancer, DPD activity had to be tested in this patient. A first uracilemia measurement return a value of 85.3 ng/mL, leading to a partial DPD deficiency. UH_2_/U ratio was equal to 3.9 (UH_2_ being equal to 332.7). Glomerular filtration rate according to CDK-EPI was 91.5 mL/min/1.73m², total bilirubinemia was 6 mg/L, GGT and ASAT 2N, and ALAT were normal. Because there were some doubts about potential pre-analytical issues potentially leading to hyperuracilemia (e.g., centrifugation done too late as compared to recommendations…), one week later, U and UH_2_ were controlled at, respectively, 82.6 ng/mL and 348 ng/mL (UH_2_/U = 4.2). Hepatic and renal biological parameters re-evaluated meanwhile were consistent with the previous values. To confirm the previous values, a third test was performed one week later, with U and UH_2_ equal to, respectively, 99.6 ng/mL and 217.1 ng/mL. The UH_2_/U ratio was, thus, a little bit lower, at 2.2. All these uracilemias were unusually high, as these values were above the 99 percentiles of the uracilemia and leaded to characterize the patient as partially deficient. Based on genotyping routinely performed in the hospital, there were no mutations known to be associated with DPD deficiency (detection of DPYD*2A, D949V, *6, *13 and HapB3 by allelic discrimination with a QuantStudio^®^ 5 Real-Time PCR system). A complete exome analysis was conducted as described previously and did not reveal any rare mutation on *DPYD* gene (18).

Because the patient was presenting a disseminated intravascular coagulation (DIC), a chemotherapy had to be rapidly started. Prothrombin time was at 44%, fibrinogen at 1.1 g/L, platelets at 131 000/L, and normal Factor V. Clinically there was spontaneous hematoma, particularly on the posterior surface of the right thigh and on the upper limb. Bleeding in the central venous line was observed. The patient did not present any thrombotic phenomenon. Thus, mid-April 2022, a chemotherapy by docetaxel 50 mg/m² and oxaliplatin 85 mg/m² was initiated.

After the first cycle, the patient’s general condition gradually improved. He started oral feeding (since then, he was fed by nasogastric tube). No recurrence of bleeding, neither diarrhea was evidenced at home. The tolerance of chemotherapy was excellent without adverse events reported by the patient. Thus, 5-FU infusion (without bolus) was initiated at the second cycle at 20% of the recommended dose (i.e., 800 mg over 46h). A blood sample was drawn 13h54 after the beginning of 5-FU to measure the exposure. The AUC was equal to 4 mg.h/L (N: [20-30 mg.h/L]).

**Figure 1:**
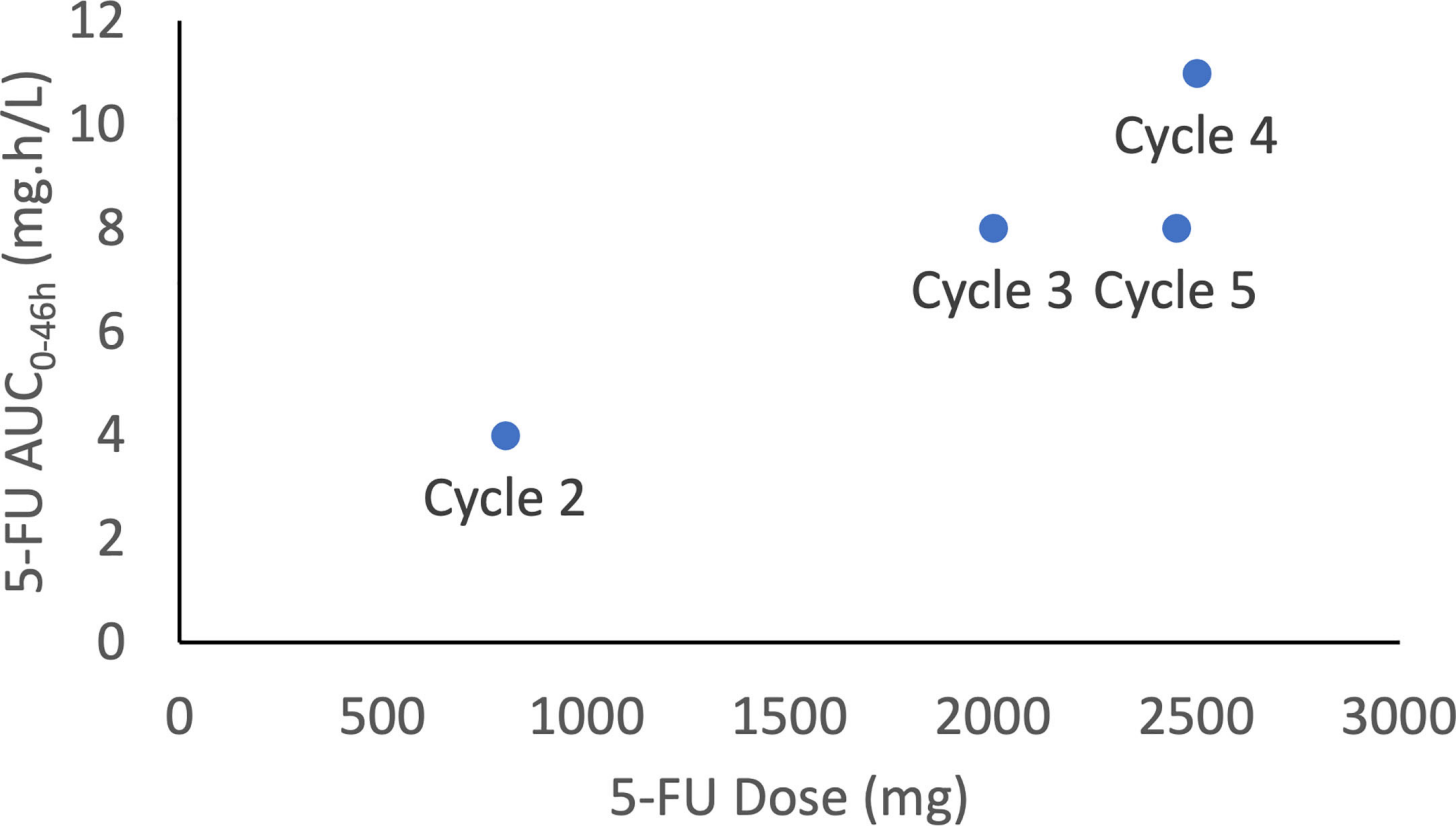
5-FU exposure (AUC_0-46h_) vs. 5-FU dose for the 4 cycles where 5-FU was dosed.

5-FU dose was thus increase up to 2500 mg over 46h (**Figure 1**). In addition to asthenia, the only side effect was grade 1 diarrhea, leading to an interruption in the dose rising. After cycle 5, the patient was then switched to tipiracil/trifluridin, but died one week later due to intracerebral hemorrhage, independent of his pathology.

Thus, in this specific situation, without Therapeutic Drug Monitoring (TDM), our patient would never have received 5-FU because of the risk of over-exposure linked to its DPD deficiency.

## Discussion

To the best of our knowledge, this is the first case report where 5-FU is safely dosed in a patient with a known relatively important partial deficiency.

Our first problematic was to ensure that this deficiency was a real one. Indeed, recent papers have shown that uracilemia may be artificially increased due to non-controlled pre-analytical conditions, renal or hepatic impairment (19–22). However, no prior organ dysfunction was evidenced in our patient. Moreover, despite doubts on the pre-analytical handling of the 1^st^ uracilemia measurement, the two other samples were closely monitored and confirmed the deficiency. Thus, even in the absence of any mutation on *DPYD* gene (by RT-PCR and exome analyses), the DPD deficiency harbored by the patient was a real deficiency.

The second problematic was then to evaluate the dose of 5-FU that could be given. None of the key recommendations on DPD deficiency handling suggest a refined 5-FU dosing strategy based solely on uracilemia values. Additionally, Dolat et al. has shown the absence of correlation between uracilemia and 5-FU clearance for uracilemia up to 30 ng/mL (23). However, in the French guidelines, TDM is proposed as an option to dose-adapt 5-FU in case of deficiency (13). As 5-FU TDM is routinely conducted in our institution, and because of the need to quickly start an effective chemotherapy in our patient, we have decided to start with a very low dose of 5-FU, estimate the exposure by TDM approach and increase doses accordingly. This strategy allowed to increase doses up to 50% of the nominal dose, with only limited toxicity. Unfortunately, at the highest tested dose, the patient remained under-exposed, which could have led to a potential lack of efficacy. This important point highlight one of the limitations of adapting 5-FU dose solely on uracilemia: deficient patients are at high risk of underexposure and the lack of efficacy may be life-threatening.

5-FU TDM should be done during the 1^st^ 5-FU controlled flow infusion (not during bolus and avoid gravity diffusers). The 1^st^ dose must be adapted to DPD phenotype or *DPYD* genotype. A single 3 mL blood sample collected in heparinized tube, without separator gel, at least 4 h after the start of the perfusion, in the morning between 8 and 10 a.m., is enough. Centrifugation should be done maximally 30 min after sampling, or a stabilizer reagent (gimeracil) must be added to the blood sample just after sampling (maximal delay before centrifugation: 24 h). AUC is calculated by multiplying the 5-FU concentration by the infusion duration and compared to an algorithm (24).

This strategy could, consequently, be adapted to other patients with known DPD deficiency, in particular in case of discrepancy between genotype and phenotype, such as for our patient. As DPD phenotyping might leads to eventual underexposure (25), we think that TDM associated to 5-FU tolerance during previous cycles may help to recover an optimal 5-FU dose. However, 5-FU introduction should be conducted by experts with the support of a multidisciplinary team and only if no therapeutic alternative is available.

## Data availability statement

The raw data supporting the conclusions of this article will be made available by the authors, without undue reservation.

## Ethics statement

Written informed consent was obtained from the participant/patient(s) for the publication of this case report.

## Author contributions

FG participated in the treatment of this case. AS drafted the manuscript. BR and JB participated in measurement of uracilemia and 5-fluorouracil concentrations. RB was responsible of the pharmacogenetic analysis. All authors contributed to the article and approved the submitted version.
